# Ticking off the Tick Vectors: *Rhipicephalus microplus* Fails to Transmit *Theileria orientalis*

**DOI:** 10.3390/pathogens12111311

**Published:** 2023-11-03

**Authors:** Abdul Ghafar, Alejandro Cabezas-Cruz

**Affiliations:** 1 Melbourne Veterinary School, The University of Melbourne, Werribee, VIC 3030, Australia; 2 ANSES, INRAE, Ecole Nationale Vétérinaire d’Alfort, UMR BIPAR, Laboratoire de Santé Animale, F-94700 Maisons-Alfort, France

*Theileria* (*T*.) *orientalis*, a tick-borne haemoparasite of cattle, is an important cause of non-transforming theileriosis in Australasia, southeast Asia, and the United States [1,2,3]. Clinically affected cattle often display symptoms such as anaemia, hypoxia, weakness, and increased cardio-pulmonary rates [4,5]. The taxonomy of *T. orientalis* has been a subject of debate, with three previously proposed species: *T. buffeli* (Australia), *T. sergenti* (Japan), and *T. orientalis* (Europe and elsewhere) [6]. However, recent evidence from PCR-directed sequencing and phylogenetic analyses of major piroplasm surface protein (*MPSP*) sequences has confirmed that they all belong to the same *T. orientalis* species, comprising 11 distinct allelic types, including the Ikeda type typically associated with clinical disease [1,2,3].

Recent associations of Ikeda type *T. orientalis* with clinical disease outbreaks in cattle herds in Australia (since 2011), New Zealand (since 2012), and the USA (since 2017) indicate that naïve herds are at increased risk of production losses due to high morbidity and mortality [2,3]. To date, *Haemaphysalis* (*H*.) *longicornis* is the only confirmed tick vector for *T. orientalis*. However, some other tick species, such as *Rhipicephalus* (*R*.) *microplus* [7], and various mechanical vectors, have been suggested to play a role in its transmission [2,8]. Given the invasive spread of *H. longicornis* ticks across more than 18 states in the USA since its first report in 2017 in New Jersey [9,10], naïve cattle herds are at an increased risk of potential pathogens this tick species may carry and transmit. In addition, it is essential to consider that the transmission of tick-borne pathogens is a complex process, and multiple tick species can serve as vectors for the same or different pathogens in the same or different host species. Therefore, it is crucial to investigate whether other native tick species might also play a role in the co-transmission of pathogens like *T. orientalis*?

In a recent study, Onzere et al. [11] investigated the potential role of *R*. *microplus* ticks as vectors for *T. orientalis* Ikeda. Animal transmission experiments in their study demonstrated that *R. microplus* was not a competent vector for the US isolate of *T. orientalis* Ikeda [11]. Moreover, this study showed that *T. orientalis* is not transmitted through transstadial and transovarial routes in *R. microplus* [11]. These findings are significant, especially considering prior empirical evidence that has confirmed the role of *R. microplus* as a vector for the closely related apicomplexan pathogens (*T. equi*, *Babesia bovis*, and *Babesia bigemina*) in equine and cattle [12,13,14]. Furthermore, *T. orientalis* DNA has been detected in field-collected *R. microplus* ticks from various countries, and as a member of the *Rhipicephalus* genus, *R. appendiculatus*, it is the primary vector for *T. parva*, the causative agent of East Coast fever in Africa [15]. Despite the differences in biology between *R. microplus* and *H. longicornis*, the failure to transmit *T. orientalis* in this study suggests that *R. microplus* does not play a role in the field transmission of oriental theileriosis. Nevertheless, these findings should be further investigated as they could be influenced by a number of factors such as lower parasitaemia levels, a potential dilution effect arising from tick pooling, the relatively small sample size in animal experiments, and differences in the genetics and microbiota of the tick colonies used in the study compared to field ticks.

The competence of ticks as vectors is influenced by a multitude of factors, including tick and pathogen species and strains, parasitaemia levels, host range and number, a pathogen’s capacity to bypass infection barriers within ticks (midgut, salivary glands, and innate immunity), tick microbiome–pathogen interactions, cross-immunity interference, and various abiotic factors [16]. The tick microbiota plays a pivotal role in pathogen acquisition and transmission, and any disruptions in it can significantly alter pathogen transmission [17]. 

In addition to systemic transmission, co-feeding transmission, where ticks feed closely on the same host, is an important route for pathogen acquisition and transmission [18]. Therefore, the findings presented by Onzere et al. [11] are of profound significance as they advance our understanding of disease transmission mechanisms in the context of invasive tick species and pathogens. They provide a solid foundation for further investigations that should explore the potential role of other native tick species as vectors for *T. orientalis*. Future studies should explore the interactions between the microbiome of various tick species, including *R. microplus* and *T. orientalis*. Moreover, animal transmission experiments should be conducted using potential vector tick species in natural environments and co-feeding as a known route for pathogen transmission from tick to tick. 

With increasing global trade and transportation, the risks of biological invasions involving arthropods and pathogens have grown considerably. Therefore, such research investigations will not only enhance our understanding of tick species acting as vectors for pathogens but will also contribute to improving cattle health outcomes in the event of biological invasion incidents caused by ticks and tick-borne pathogens. These investigations will help improve the surveillance of potential spill-over events to and from native tick species, facilitating the design of improved control measures through a deeper understanding of the presence and abundance of other potential tick vectors.
